# Icebergs in the Nordic Seas Throughout the Late Pliocene

**DOI:** 10.1002/2017PA003240

**Published:** 2018-03-30

**Authors:** Y. M. Smith, D. J. Hill, A. M. Dolan, A. M. Haywood, H. J. Dowsett, B. Risebrobakken

**Affiliations:** ^1^ School of Earth and Environment University of Leeds Leeds UK; ^2^ U.S. Geological Survey Reston VA USA; ^3^ Uni Research Climate Bjerknes Centre for Climate Research Bergen Norway

**Keywords:** Pliocene, paleoceanography, iceberg, Nordic Seas, modeling, ice‐rafted debris

## Abstract

The Arctic cryosphere is changing and making a significant contribution to sea level rise. The Late Pliocene had similar CO_2_ levels to the present and a warming comparable to model predictions for the end of this century. However, the state of the Arctic cryosphere during the Pliocene remains poorly constrained. For the first time we combine outputs from a climate model with a thermodynamic iceberg model to simulate likely source regions for ice‐rafted debris (IRD) found in the Nordic Seas from Marine Isotope Stage M2 to the mid‐Piacenzian Warm Period and what this implies about the nature of the Arctic cryosphere at this time. We compare the fraction of melt given by the model scenarios with IRD data from four Ocean Drilling Program sites in the Nordic Seas. Sites 911A, 909C, and 907A show a persistent occurrence of IRD that model results suggest is consistent with permanent ice on Svalbard. Our results indicate that icebergs sourced from the east coast of Greenland do not reach the Nordic Seas sites during the warm Late Pliocene but instead travel south into the North Atlantic. In conclusion, we suggest a continuous occurrence of marine‐terminating glaciers on Svalbard and on East Greenland (due to the elevation of the East Greenland Mountains during the Late Pliocene). The study has highlighted the usefulness of coupled climate model‐iceberg trajectory modeling for understanding ice sheet behavior when proximal geological records for Pliocene ice presence or absence are absent or are inconclusive.

## Key Point

- In the Nordic Seas, evidence suggests icebergs have persisted throughout the Late Pliocene
- A coupled climate model‐iceberg trajectory model can prove useful for understanding paleo‐ice sheet behavior
- Ice appears to have persisted on Svalbard and East Greenland throughout the Late Pliocene

## 1 Introduction

The Pliocene epoch (5.33 Ma to 2.58 Ma) is the last interval of Earth history that had temperatures similar to those expected by the end of the 21st century (Collins et al., 2013). However, within the Late Pliocene (Piacenzian Stage; 3.60–2.58 Ma) proxy records show fluctuations from glacial to interglacial conditions (e.g., De Schepper et al., 2014). The mid‐Piacenzian Warm Period (mPWP; 3.264–3.025 Ma) is a particularly well‐studied interval of the Pliocene (Dowsett, 2007; Dowsett et al., 2010, 2016; Haywood et al., 2011; Haywood, Hill, et al., 2013). During this period, CO_2_ levels were similar to present day (~400 ppmv) (Pagani et al., 2010; Seki et al., 2010). Simulations suggest that the mPWP had a warmer and wetter climate, with global annual mean surface temperatures 1.86 to 3.46°C warmer than present (Haywood, Dolan, et al., 2013). While Bachem et al. (2016) showed that sea surface temperatures (SST) along the coast of Norway reached up to 14°C, SSTs in the Nordic Seas show a great deal of variability among results (4°C to 19°C) depending on the proxy used (e.g., Knies, Cabedo‐Sanz, et al., 2014; Robinson, 2009; Schreck et al., 2013). Vegetation shows a northward shift of the taiga‐tundra boundary (Salzmann et al., 2009, 2011) with evidence of cool temperate (deciduous to mixed) forest pollen from the coast of Norway at Ocean Drilling Program (ODP) Hole 642B (Panitz et al., 2016). This is in contrast to the mainly boreal forest and peatlands present today (Moen, 1987). In Arctic regions, evidence of coniferous tree material has been found at Île de France, northeast Greenland, and the Beaver Pond locality, Ellesmere Island, Canada (Bennike et al., 2002; Matthews Jr. & Ovenden, 1990). The latitudes of Île de France and Beaver Pond are located at 77.44°N and 76.26°N, respectively. To put these locations into context, the latitudinal extent of coniferous forest at present is between 50°N and 60°N (Taggart & Cross, 2009).

Marine Isotope Stage (MIS) M2 is identified as a large increase in benthic δ^18^O (3.74‰) at approximately 3.3 Ma (Lisiecki & Raymo, 2005). Although this is a large departure from the trend and has been suggested to document the intensification of glaciation during the Pliocene (Haug & Tiedemann, 1998), there exists an enigma as there are several sampled sites which do not show the same magnitude of change. Although there is evidence for localized glaciers/ice caps in the Northern Hemisphere during MIS M2, there is limited terrestrial evidence to indicate a large‐scale glaciation (Dolan et al., 2015, and references therein; see also De Schepper et al., 2014). Lunt et al. (2008) tested various hypotheses for the onset of Northern Hemisphere glaciation at 2.75 Ma and identified a drop in CO_2_ as the most likely mechanism to account for ice growth. Bartoli et al. (2011) suggested a CO_2_ level of approximately 220 ppmv during MIS M2. The large amplitude of the shifts (about 0.5‰ in δ^18^O, Lisiecki & Raymo, 2005), far‐field sea level records (65 m ± 15 to 25 m, Dwyer & Chandler, 2009; 10 m ± 10 to 15 m, Naish & Wilson, 2009), and global sea level records (Miller et al. (2005, 2011, 2012); 40 m ± 10 m) suggest the potential for a major sea level fall across MIS M2. This implies a buildup of continental ice sheets in the Northern Hemisphere, an expansion of Southern Hemisphere ice, or both. Dolan et al. (2015) prescribed different idealized ice sheet configurations and found that surface climatic conditions with a large Northern Hemisphere ice sheet were not inconsistent with available proxy records.

Alongside climatological evidence for the warm Late Pliocene and MIS M2, there is evidence of ice‐rafted debris (IRD) deposited in the Nordic Seas (Bachem et al., 2016; Fronval & Jansen, 1996; Jansen et al., 2000; Knies, Mattingsdal, et al., 2014) implying the potential existence of marine‐terminating glaciers within this region. However, the source regions for deposited IRD remain unclear. By comparing the modeled trajectories with IRD records (and the variability they show), it is possible to infer where marine‐terminating glaciers have persisted during the MIS M2 and mPWP. This model‐data comparison also has the potential to inform our understanding of the extent of Northern Hemisphere ice and in particular the Greenland Ice Sheet during the Late Pliocene.

**Figure 1:**
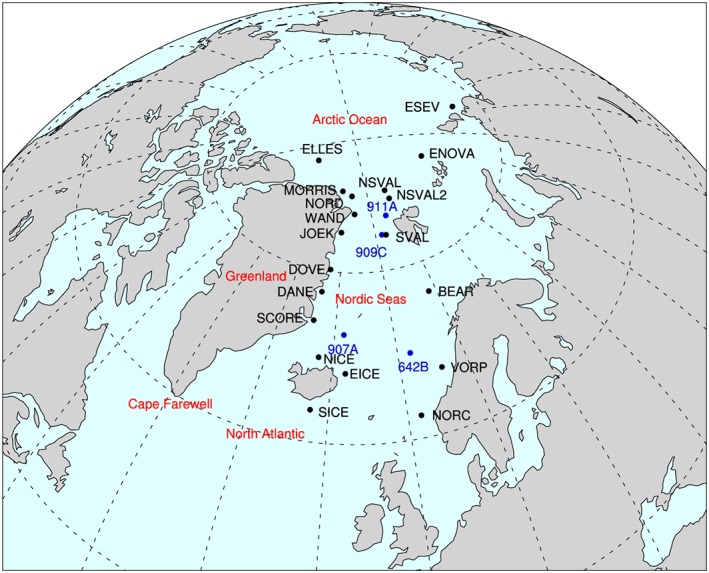
Map of the Nordic Seas Ocean Drilling Program sites and the surrounding seeding locations. Ocean Drilling Program sites are marked in blue. Seeding locations are marked in black. NORC = Norwegian Channel; VORP = Vøring Plateau; BEAR = Bear Island; SVAL = Svalbard; NSVAL = northern Svalbard; NSVAL2 = northern Svalbard 2 (see text); ENOVA = eastern Novaya Zemlya; ESEV = eastern Severnaya Zemlya; ELLES = Ellesmere Island; MORRIS = Morris Jessup; NORD = Nord; WAND = Wandell Sea; JOEK = Joekelbugt; DOVE = Dove; DANE = Daneborg; SCORE = Scoresby Sund; NICE = northern Iceland; EICE = eastern Iceland; SICE = southern Iceland.

Here we analyze iceberg trajectory modeling results that use scenarios derived from climate modeling studies to calculate the total melt of icebergs from different seeding locations around the Nordic Seas (Figure 1). The iceberg trajectories and fraction of melt is compared to synthesized IRD records from ODP sites within the Nordic Seas (see section 2.3 and Figure 1). We discuss the combined model and data information to advise on most likely source areas for the IRD found at the different Nordic Seas ODP sites from MIS M2 to the mPWP.

## 2 Methods

### 2.1 Climate Model Boundary Conditions and Experimental Design

The climate model HadCM3 (Gordon et al., 2000) was used to provide the climate and ocean circulation parameters required by the iceberg trajectory model. The climate model was set up to produce three different climate scenarios (Table 1). The warm Pliocene (mPWP_STD_) and the altered paleogeography (mPWP_ALT_) scenario conditions were those used within Pliocene Model Intercomparison Project Phase 1 (PlioMIP) (Haywood et al., 2011) and used a CO_2_ level of ~ 405 ppmv (Hill, 2015). The difference between the mPWP_STD_ and the mPWP_ALT_ scenarios is the geography only (Table 1). For the representation of the cold MIS M2 (M2) scenario, the required environmental and ocean circulation parameters were derived from the MIS M2 climate modeling study of Dolan et al. (2015) and in particular the large ice configuration. Key geographic changes in mPWP_ALT_ are already seen in MIS M2 because of the drop in sea level, and so a MIS M2 with altered paleogeography was not undertaken. This study uses the most extreme MIS M2 scenario of Dolan et al. (2015) to look at greatest possible change using a covered Barents Sea to provide the largest sea level drop. Uncertainties exist to the location and extent of ice sheets within the Northern Hemisphere during the Pliocene, and a part of this study is to aid in the identification of such. Further details of specific parameters are found in Table 1.

**Table 1:** Experiment Notation Used in This Paper With Description and Reference

Climate	Description	CO_2_	Ice sheet	Vegetation	Paleogeography	Citation
M2	Cold MIS M2 scenario	220 ppmv (Bartoli et al., 2011)	Large ice mass over Greenland and North America; small ice mass on Scandinavia	Dynamically derived	Modern topography outside of ice sheet areas based on Standard HadCM3 modern	Dolan et al. (2015)
mPWP_STD_	Mid‐Piacenzian Warm Period scenario	405 ppmv (Pagani et al., 2010; Seki et al., 2010)	Small ice mass on the East Greenland highlands (Hill et al., 2007, 2010) PRISM 3 reconstruction (Dowsett et al., 2010)	Based on Salzmann et al. (2008)	Topography based on Sohl et al. (2009)	Hill (2015); see also Bragg et al. (2012)
mPWP_ALT_	Mid‐Piacenzian Warm Period scenario with an altered paleogeography	405 ppmv (Pagani et al., 2010; Seki et al., 2010)	Small ice mass on the East Greenland highlands (Hill et al., 2007, 2010) PRISM 3 reconstruction (Dowsett et al., 2010)	Based on Salzmann et al. (2008)	Subaerial Barents Sea (Butt et al., 2002); increased depth of Greenland‐Scotland Ridge (Wright & Miller, 1996); North America rivers (MacKenzie, Ohio, Missouri, and St Lawrence and rivers currently flowing into the hudson Bay) rerouted to flow into the Labrador Sea at the Hudson Strait (Duk‐Rodkin & Hughes, 1994; Mack et al., 2006; Prather, 2000); Baltic Basin rivers routed to a reinstated Eridanos River (Overeem et al., 2001)	Hill (2015); see also Bragg et al. (2012)

The term mPWP is used to differentiate between discussion about the mPWP in general terms and when talking about the mPWP climate scenario which uses the standard PlioMIP paleogeography (mPWP_STD_) and the altered paleogeography (mPWP_ALT_) of Hill (2015).

### 2.2 Iceberg Modeling

The Bigg et al. (1997) thermodynamic iceberg model uses the climatological data provided by the HadCM3 climate model to drive icebergs released from various locations (henceforth referred to as seeding locations) around the perimeter of the Nordic Seas. At these locations, icebergs were seeded into the model which has the capacity to ground and flip them creating a more realistic life span of the iceberg. Several factors affect the horizontal speed of an iceberg, including water drag, air drag, wave radiation force, and horizontal pressure gradient force of the displaced water. These are combined with iceberg mass and Coriolis force to give a formula for horizontal motion (Bigg et al., 1997). The dominant force transporting icebergs is the ocean current; however, wind, in combination with ocean current, has a greater influence on trajectories than the ocean current in combination with other individual forces (Matsumoto, 1997). The iceberg model uses icebergs of differing size classes based on those of Gladstone et al. (2001) which circulate in the ocean until they have fully melted. The length of these melt periods take into account iceberg grounding in shallow waters until they are melted sufficiently to move once more. All these factors within the model are important in order to produce the most accurate iceberg trajectory. IRD is not tracked within the iceberg model as there is no sediment loading within the model. Therefore, it is assumed that the meltwater fraction can be used to approximate where IRD could melt out of an individual iceberg.

The location of the Pliocene drainage basins for all these areas is still an unknown, and so an exhaustive number of sites were used to model the iceberg trajectories. The iceberg seeding locations were informed by Bigg et al. (1997) and Ottesen et al. (2005). Bigg et al. (1997) examined bathymetric, glacial, and geological maps of the Arctic Ocean, Greenland, and Canadian Arctic and identified the locations of marine‐terminating glaciers, ice shelves, and ice caps. Identified sites that were close together were combined due to the 1° resolution of the driving ocean model. The Nordic locations were based on Ottesen et al. (2005) who identified the Bear Island Trough Ice Stream and the Norwegian Channel Ice Stream to be the two largest according to analysis of seafloor geophysical features. Ottesen et al. (2005) also identified several areas along the coast of Norway between these two largest streams and also on west coast Svalbard where megascale lineations indicate large glacial drainage basins. The location of marine‐terminating glaciers throughout the study area during the Pliocene is unknown, and so although Bigg et al. (1997) and Ottesen et al. (2005) refer to the modern and Quaternary, respectively, these sites are prolific and give a good indication to the differences in trajectories of icebergs due to seeding location. An arbitrary number of 100 icebergs of differing mass (6.113 × 10^8^ to 6.657 × 10^11^ kg) were released each model month from several locations around the Nordic Seas and Arctic Ocean (Figure 1). These seeding locations have been grouped into Arctic locations (eastern Severnaya Zemlya (ESEV), eastern Novaya Zemlya (ENOVA), northern Ellesmere Island (ELLES), and northern Svalbard (NSVAL and NSVAL2)), Greenlandic locations (Nord (NORD), Morris Jessup (MORRIS), Wandell Sea (WAND), Joekelbugt (JOEK), Dove (DOVE), Daneborg (DANE), and Scoresby Sund (SCORE)), Icelandic locations (northern Iceland (NICE), southern Iceland (SICE), and eastern Iceland (EICE)), and Nordic locations (Norwegian Channel (NORC), east of the Vøring Plateau (VORP), Bear Island (BEAR), and southwest Svalbard (SVAL)). A site along the north of Svalbard (NSVAL) at latitude 83.1°N was seeded during the M2 scenario. During the mPWP_STD_ and mPWP_ALT_ scenarios, a site closer to the mainland of the island was seeded with icebergs at latitude 80.4°N (NSVAL2; Figure 1). This site was the only site which was subject to such large changes in the landmass in the model due to the fact that the mPWP_STD_ model does not include a landmass for Svalbard and the mPWP_ALT_ model includes a subaerial Barents Sea.

### 2.3 IRD Data

Previously published Nordic Seas IRD records that cover MIS M2 to mPWP, from ODP Holes 911A, 907A, and 642B (Bachem et al., 2016, 2017; Fronval & Jansen, 1996; Jansen et al., 1990; Knies, Mattingsdal, et al., 2014) have been compiled and are presented together with new IRD occurrence (presence/absence) data from ODP Holes 911A, 909C, 907A, and 642B. All records from any site are presented using the same chronological framework (see Table 2).

**Table 2:** Location of Investigated ODP Holes and Information on the Chronological Constraints Behind the Age Models Used

ODP hole	Location	Latitude	Longitude	Depth (mbsl)	Reference for age model	Basis for age constraints	Data reference
642B	Vøring Plateau	67°13.5′N	2°55.7′E	1,292.7	Risebrobakken et al. (2016)	Paleomagnetic reversals; LR04	Jansen et al. (1990) https://doi.pangaea.de/10.1594/PANGAEA.882973 Bachem et al. (2016) https://doi.org/10.1594/PANGAEA.858944 Bachem et al. (2017) https://doi.org/10.1594/PANGAEA.865214
907A	Iceland Plateau	69°14.989′N	12°41.894′W	1,800.8	Jansen et al. (2000)	Paleomagnetic reversals; orbitally tuned ice‐rafted debris	Fronval and Jansen (1996) https://doi.org/10.1594/PANGAEA.848081
909C	Hovgård Ridge	78°35.096′N	3°4.222′E	2,517	Robinson (2009)	Paleomagnetic reversals	This study https://doi.pangaea.de/10.1594/PANGAEA.884332
911A	Yermak Plateau	80°28.466′N	8°13.640′E	901.6	Mattingsdal et al. (2014)	Paleomagnetic reversals; biostratigraphy (foraminifera and palynological studies)	Knies, Cabedo‐Sanz, et al. (2014) https://doi.pangaea.de/10.1594/PANGAEA.883471

*Note*. References to the papers where the age models were originally published and where further details on the establishment of these age models used can be found are given. Furthermore, references to where the data were first published and where they are available online are given. ODP = Ocean Drilling Program.

The previously published IRD record from ODP Hole 911A is presented as weight percent of sediment in the 0.1 to 1 mm fraction of the samples (Knies, Mattingsdal, et al., 2014). The published records from ODP Holes 907A and 642B are all presented as number of IRD grains per gram sediment. The record from 907A is counted as number of IRD grains per gram sediment >125 μm (Fronval & Jansen, 1996). For Hole 642B, two published IRD records exist for the investigated interval. The record from Jansen et al. (1990) presents number of IRD grains per gram sediment >125 μm, while the higher‐resolution record from Bachem et al. (2016) presents number of IRD grains per gram sediment >150 μm. Since different methods have been used in the original studies to establish the IRD records, the absolute values are not directly comparable between the sites, with the exception of the >125 μm records from ODP Holes 907A and 642B of Fronval and Jansen (1996) and Jansen et al. (1990), respectively. The patterns of variability are, however, comparable between the locations. Furthermore, the presence or absence of IRD within each record provides true information about changes in IRD deposition at each location at different times through the investigated time interval. Thus, IRD records can be compared to the presence of meltwater found at the locations for the different seeding and climate scenarios investigated by the thermodynamic iceberg model.

The new occurrence data are obtained from ODP samples made available through the U.S. Geological Survey PRISM (Pliocene Research Interpretation and Synoptic Mapping) Project (Dowsett et al., 2015). These samples initially consisted of 10–20 cm^3^ of sediment washed over a 63 μm or 150 μm mesh (full details of this process can be found in Dowsett et al., 2015). The ≥150 μm residue was viewed under a light microscope, and occurrence (presence or absence) of IRD was registered (Figure 5). Before this study, no information existed about occurrence of IRD in Hole 909C over MIS M2 to the mPWP; hence, the occurrence data add information from one site to the compilation.

In addition to the Pliocene IRD data described above, we show a previously unpublished IRD data set (number of grains ≥150 μm/g sediment) covering the last interglacial from the Vøring Plateau core MD95‐2010 (66°31.05 N, 04°33.97′E, 1,226 m water depth; Risebrobakken et al., 2005). This record is presented for comparison of the level of IRD deposited at the Vøring Plateau during the Pliocene with a record from the same area and a warm climate state when the constraints on ice sheet extent are better known.

## 3 Results

Here we present results using three different Pliocene climate scenarios: MIS M2 (M2), warm Pliocene (mPWP_STD_), and warm Pliocene with altered paleogeography (mPWP_ALT_); see Table 1 for further details. A preindustrial simulation was run to enable the calculation of the anomaly in climatological fields. Initially we describe the climatological fields that are relevant in the context of the iceberg trajectory modeling. We present the simulated iceberg trajectories and meltwater fractions for each model scenario, as well as IRD data from the four ODP sites in the Nordic Seas.

### 3.1 Modeled Climatological States in the Nordic Seas

#### 3.1.1 Ocean Circulation in the Nordic Seas

**Figure 2:**
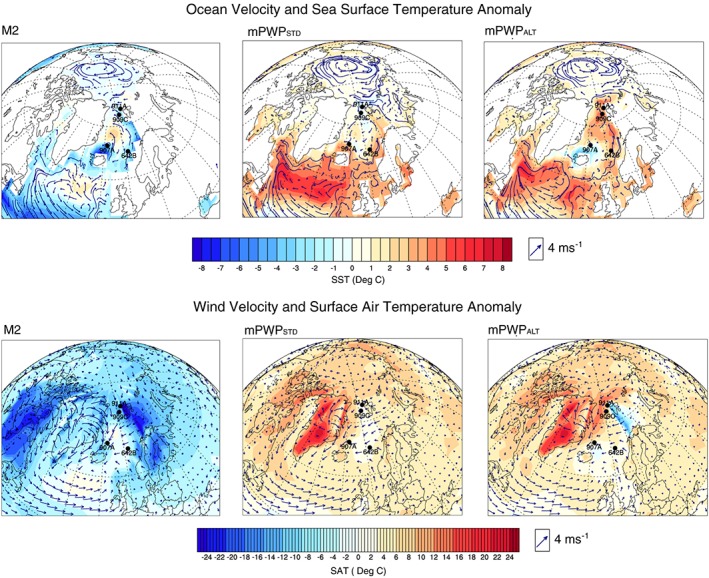
Climatological boundary conditions of the M2, mPWP_STD_, and mPWP_ALT_ scenarios. Ocean and wind currents are the two main drivers of iceberg trajectories; however, sea surface temperatures (SST) and surface atmospheric temperature (SAT) influence the melt rate. The SST and SAT anomalies are calculated using annual mean temperatures. The wind velocity scale arrow is measured at 4 m s^−1^. M2 = Marine Isotope Stage M2; mPWP_STD_ = standard mid‐Piacenzian Warm Period; mPWP_ALT_ = mid‐Piacenzian Warm Period scenario with an altered paleogeography.

In the modeled M2 scenario, the Norwegian‐Atlantic Current splits north of the Faroe Islands with a branch heading west around the northern coast of Iceland and the other branch flowing north along the coast of Norway, western Barents Sea, and west Svalbard. In the mPWP_STD_ and mPWP_ALT_ scenarios, the model‐predicted circulation in the Nordic Seas (Figure 2) shows a strong northerly Norwegian‐Atlantic Current across the Iceland‐Scotland Ridge, along the coast of Norway, the western boundary of the Barents Sea, and west Svalbard. In the mPWP_STD_ scenario, and to a lesser extent the mPWP_ALT_ scenario, a distinct East Greenland Current (EGC) completes the anticlockwise circulation pattern.

#### 3.1.2 Sea Surface Temperature

In the modeled M2 scenario the Norwegian Sea varies from 0 to 5°C cooler than preindustrial. The mean annual temperature is coldest closest to land and warms by 3 to 4°C in the center of the Nordic Seas. Around Iceland the mean annual SST is 1°C cooler on the southeastern coast and 8°C cooler on the northwestern coast. Along the coast of Greenland the difference in temperature from present is minimal (−1°C) north of approximately 70°N but increases to −3°C through the Denmark Strait and south to Cape Farewell. The SST anomaly for the mPWP_STD_ scenario relative to the preindustrial shows a 3 to 4°C mean annual warming in the Norwegian Sea; however, along the western boundary with the Barents Sea this is approximately 1°C cooler (Figure 2). Coastal regions of Iceland are approximately 3 to 5°C warmer. In the mPWP_ALT_ scenario, this is reversed, with the coast of Iceland experiencing 3°C cooler temperatures and the western Barents approximately 5°C warmer temperatures. The majority of the Nordic Sea including the eastern coast of Greenland is 0 to 2°C (0 to 4°C in the mPWP_ALT_ scenario) warmer.

#### 3.1.3 Wind Circulation and Surface Atmospheric Temperature

In the M2 scenario winds travel north from the UK but turn westward when they meet winds traveling westward across the Nordic Seas from Norway (Figure 2). These winds meet stronger winds coming from the interior of Greenland before turning southward. In the mPWP_STD_ scenario, although the pattern is similar, the winds from Norway and those moving from the Greenland interior are weaker than those of the M2 scenario. In the mPWP_ALT_ scenario winds are similar but stronger moving southwest out of the Arctic Ocean.

During the M2 scenario, the anomaly of warm mean annual surface atmospheric temperatures (SATs) seen along the Norwegian coast are replaced with cooler temperatures of 0 to −10°C along the southern coast of Norway, dropping to −30°C toward the Arctic Ocean. In the Greenland interior, the mean annual SAT anomaly drops to −45°C. In the mPWP_STD_ scenario, a SAT anomaly of between 3 to 6°C extends from the North Atlantic up the coast of Norway to the western boundary of the Barents Sea. The central Nordic Seas from eastern Iceland to western Svalbard have a mean annual SAT between 0 and 3°C warmer than present. SATs along the east Greenland coast range between 3 and 12°C warmer than present. In the mPWP_ALT_ scenario, SATs along the Norwegian coast are 0 to 3°C warmer than present with a colder (up to 15°C) SAT anomaly along the west Barents margin. North of Iceland cooler than present SATs exist (0 to 3°C), but along the east coast of Greenland SATs reach a high of 15°C warmer than present.

#### 3.1.4 Sea Ice

**Figure 3:**
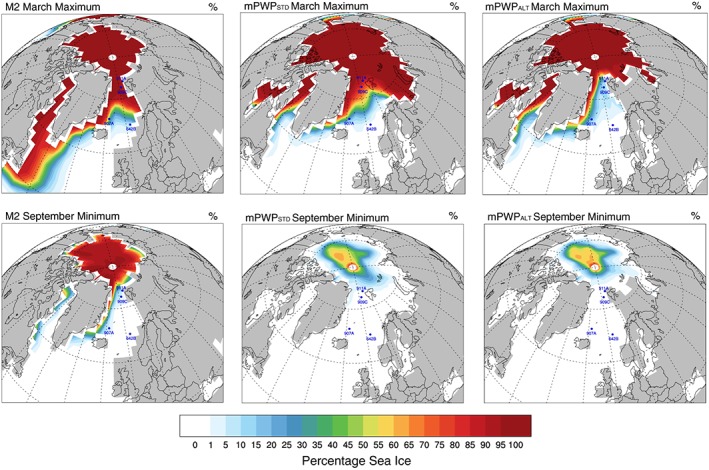
Extent and percentage concentration of sea ice. (top row) The March maximum extent of sea ice and (bottom row) the September minimum. M2 = Marine Isotope Stage M2; mPWP_STD_ = standard mid‐Piacenzian Warm Period; mPWP_ALT_ = mid‐Piacenzian Warm Period scenario with an altered paleogeography.

The extent and percentage concentration of sea ice cover varies between seasons with the summer minimum (September) and winter maximum (March) averages shown in Figure 3. During the cold M2 scenario, summer sea ice is shown to reach the coastlines around the Arctic Ocean but also extends south along the east Greenland coast to the Denmark Strait and is found along the Canadian coastline at Nares Strait and the mouth of Hudson Bay. During the winter, however, sea ice extends from the Arctic Ocean along the western boundary of the Barents Sea to the northern coast of Norway, south along the east Greenland coast and south along the east Canadian coast to Nova Scotia. During the mPWP_STD_ scenario, summer sea ice is contained within the central Arctic Ocean area. However, during the winter, the sea ice extends to the Russian coastline, Canadian archipelago, northern Scandinavia, and NSVAL in the Arctic Ocean and down the east Greenland coast to Scoresby Sund. The Baffin Sea west of Greenland has sea ice from Nares Strait to Disko Bugt. In the mPWP_ALT_ scenario, the extent is similar; however, the percentage concentration of sea ice in the Nordic Seas is much less.

### 3.2 Iceberg Modeling

**Figure 4:**
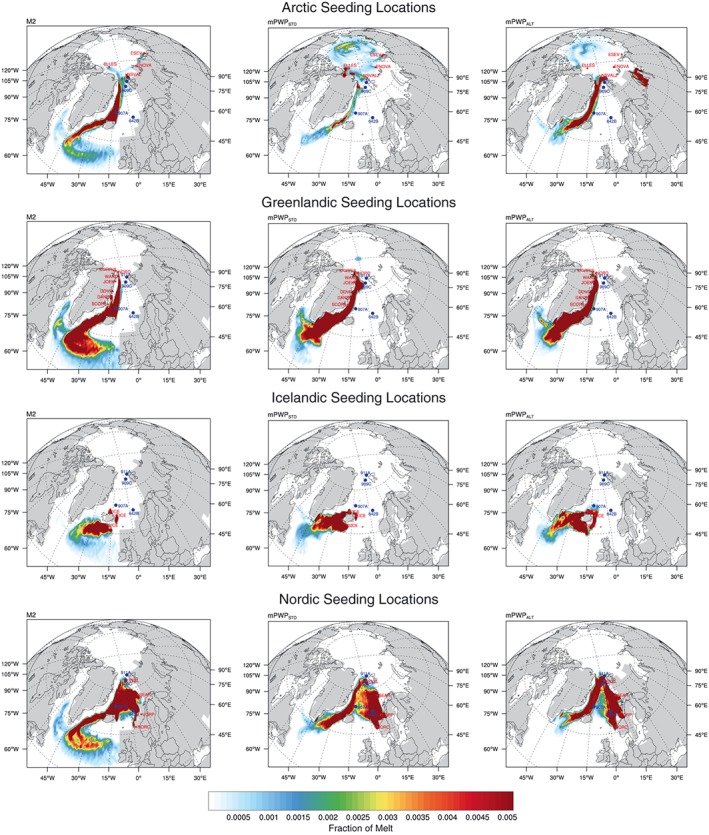
Iceberg trajectories for the 19 seeding locations for three different climate scenarios (M2, mPWP_STD_, and mPWP_ALT_). M2 = Marine Isotope Stage M2; mPWP_STD_ = standard mid‐Piacenzian Warm Period; mPWP_ALT_ = mid‐Piacenzian Warm Period scenario with an altered paleogeography.

The results of the iceberg trajectory modeling using three different climate scenarios (M2, mPWP_STD_, and mPWP_ALT_) from each of the seeding locations are grouped (as defined in section 2.2) and can be seen in Figure 4.

#### 3.2.1 Arctic Seeding Locations

Modeled icebergs seeded from locations around the Arctic Ocean (ESEV, ENOVA, ELLES, and NSVAL/2) show very different patterns between M2 and mPWP scenarios. During the M2 (Figure 4), iceberg trajectories from ELLES travel east and enter the Fram Strait before traveling southward in the EGC. Icebergs from NSVAL, ESEV, and ENOVA travel west before entering the Fram Strait and, again, entering into the EGC on their journey south. Icebergs from these seeding locations reach the Iceland Plateau and travel further south through the Denmark Strait. During the mPWP_STD_ scenario (Figure 4), modeled icebergs from ELLES travel westward, and their trajectories cover the majority of the Beaufort Basin area of the Arctic Ocean. Icebergs from ENOVA and ESEV travel westward around the perimeter of the Arctic Ocean until reaching the Canadian archipelago, where the trajectory spreads out over the Beaufort Basin area of the Arctic Ocean. Icebergs seeded from these three locations do not enter the Fram Strait. Icebergs from NSVAL2 travel across the Fram Strait and are entrained into the EGC. In the mPWP_ALT_ scenario, the subaerial Barents Sea obstructs the flow of North Atlantic water between the Norwegian coast and Svalbard and directs it northward along the Western Spitsbergen Current. With this current deflected, icebergs seeded from ENOVA in the Arctic become trapped in the Kara Sea and are not transported around the Arctic.

#### 3.2.2 Greenlandic Seeding Locations

In all the scenarios, modeled iceberg trajectories flow south in the EGC. In the M2 scenario, icebergs reach a more easterly extent in the Nordic Seas and in the North Atlantic (Figure 4). During the mPWP_STD_ and mPWP_ALT_ scenarios, icebergs do not reach across to the European coastline in the North Atlantic but rather melt in the Labrador Sea and northwestern Atlantic region. The Iceland Plateau does not appear to receive icebergs from the coast of Greenland during the mPWP_STD_ scenario, but the mPWP_ALT_ scenario does show icebergs reaching the location of 907A.

#### 3.2.3 Icelandic Seeding Locations

During the M2 scenario (Figure 4), modeled icebergs seeded from locations around Iceland do not reach ODP Hole 907A. Icebergs seeded from the northern and eastern Icelandic sites appear to melt close to their calving location. However, SICE iceberg trajectories cover the northern North Atlantic with only a small amount of icebergs heading sufficiently westward to reach the Labrador Sea. During the mPWP_STD_ scenario (Figure 4), icebergs from east Iceland melt close to their calving location. Icebergs from north Iceland enter into the Denmark Strait and melt south of Cape Farewell. Icebergs from south Iceland move westward toward the south of Cape Farewell. With the change in the depth of the Greenland‐Scotland Ridge in the mPWP_ALT_ scenario (Table 1), there also appears to be a strengthening of the current northward from the eastern coast of Iceland with icebergs from EICE reaching the Iceland Plateau.

#### 3.2.4 Nordic Seeding Locations

Modeled icebergs seeded from the coast of Norway (NORC and VORP) have very similar patterns during both the M2 and the mPWP_STD_ scenarios. In both climate scenarios, icebergs cover the entire Nordic Seas and travel south through the Denmark Strait. The difference between the two climate scenarios is the extent to which they travel after they have passed through the Denmark Strait. In the M2 scenario (Figure 4), iceberg trajectories cover the North Atlantic but also find their way into the West Greenland Current where they travel north. The icebergs then get picked up in the Labrador Current having traversed the Davis Strait and travel south where they melt in the Labrador Sea. During the mPWP_STD_ scenario (Figure 4), the icebergs melt to the east of Cape Farewell. Nordic iceberg trajectories are influenced by the altered paleogeography. In the mPWP_ALT_ scenario, their trajectories are shown to circumnavigate the Nordic Seas more than in the mPWP_STD_ scenario where they cross east to west to a greater extent.

### 3.3 Modeled Iceberg Meltwater Fraction at ODP Sites Within the Nordic Seas

Figure 4 shows the extent of iceberg melting when seeding icebergs from the Arctic, Greenlandic, Icelandic, and Nordic locations. In order to enable comparison to IRD data, we first detail the meltwater fraction seen within the modeling framework at each ODP hole.

*ODP Hole 911A*. Both the cold M2 and warm mPWP_STD_ scenarios show modeled iceberg trajectories traversing this site. The seeding locations for these icebergs are only from the margins of Svalbard. Neither icebergs from the Arctic coastline nor from further south in the Nordic Seas are able to reach the site. In the mPWP_ALT_ scenario, however, icebergs from VORP, BEAR, and SVAL reach this site.

*ODP Hole 909C*. Iceberg trajectories during the M2 scenario from seeding locations on Svalbard (NSVAL and SVAL) are the only ones modeled to reach this site. During the mPWP_STD_ scenario, icebergs from the seeding locations of Bear Island and Svalbard (BEAR and SVAL) reach this site. In the mPWP_ALT_ scenario, sites from Norway (NORC, VORP, and BEAR) and SVAL reach this site.

*ODP Hole 907A*. Iceberg trajectories reaching this site during the cold M2 scenario come from seeding locations in the Arctic Ocean (ELLES, ENOVA, ESEV, and NSVAL), east Greenland coast (MORRIS, NORD, and WAND), Svalbard and western Barents Sea (SVAL and BEAR), and off the coast of Norway (NORC and VORP). Sites from around Iceland and Scoresby Sund (EICE, NICE, SICE, and SCORE) do not appear to reach ODP Hole 907A. During the mPWP_STD_ scenario, only icebergs modeled from three locations (Norwegian Channel (NORC), west Norwegian coast (VORP) and Bear Island (BEAR)) reach ODP Hole 907A. However, in the mPWP_ALT_ scenario, icebergs from Arctic locations (SVAL and NSVAL2), east coast Greenland (WAND, NORD, and MORRIS), east Iceland (EICE), and Nordic locations (NORC, VORP, and BEAR) all reach 907A.

*ODP Hole 642B*. Icebergs from most seeding locations are unable to reach this site as it is covered by the strong northward flowing Norwegian Current. However, in the M2 scenario, modeled iceberg trajectories that can reach this site come from the Vøring Plateau and Bear Island, while in both the mPWP_STD_ and mPWP_ALT_ climate scenarios only Norwegian seeding locations NORC and VORP reach this site.

### 3.4 IRD at ODP Holes Within the Nordic Seas

**Figure 5:**
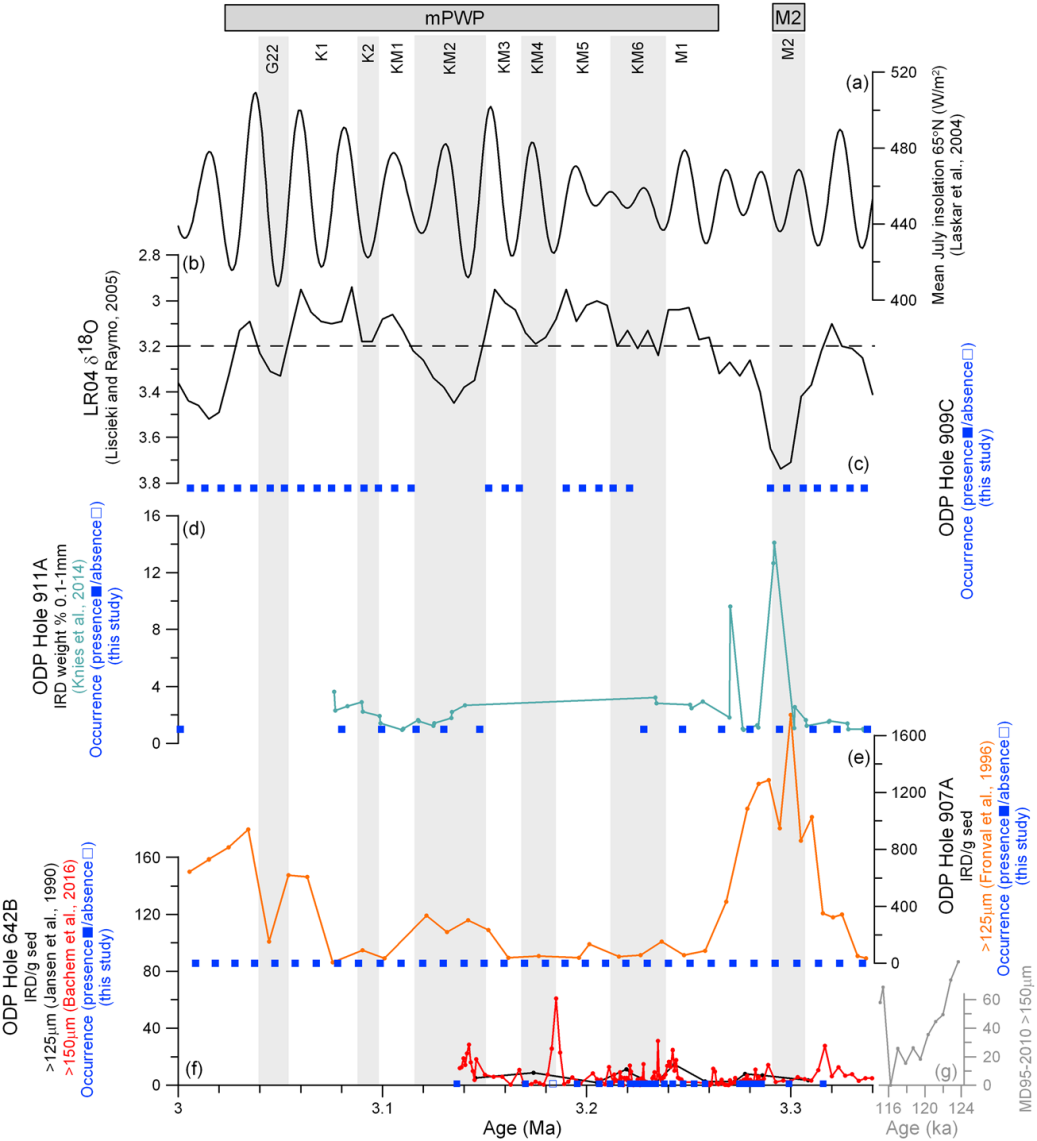
Ice‐rafted debris (IRD) compilation for the Nordic Seas. Note different scales on the *y* axes. (a) July insolation at 65°N (Laskar et al., 2004). (b) LR04 stack of benthic δ^18^O with the current δ^18^O level of 3.23‰ shown as a dashed line (modified from Lisiecki & Raymo, 2005). (c) New occurrence data showing the presence (■) or absence (□) of IRD at Ocean Drilling Program (ODP) Hole 909C. For panels (d)–(f), occurrence data are also plotted in the same manner. (d) ODP Hole 911A IRD record (weight percentage of the 0.1 to 1 mm size fraction (light blue; Knies, Mattingsdal, et al., 2014)). (e) ODP Hole 907A IRD record (number of grains >125 μm per gram sediment (orange; Fronval & Jansen, 1996)). (f) ODP Hole 642B IRD record (number of grains >125 μm (black; Jansen et al., 1990) and >150 μm (red; Bachem et al., 2016, 2017)). (g) MD95‐2010 IRD record covering the last interglacial (number of grains >150 μm (gray)). The horizontal bars denote the mid‐Piacenzian Warm Period (mPWP) (Dowsett et al., 2010) and Marine Isotope Stage M2 (MIS M2) (De Schepper et al., 2009). Vertical light gray bars identify colder isotope stages (Lisiecki & Raymo, 2005).

Figure 5 shows our compilation of published (Bachem et al., 2016; Bachem et al., 2017; Fronval & Jansen, 1996; Jansen et al., 2000; Knies, Mattingsdal, et al., 2014) and new IRD occurrence data sets (presence/absence). Overall, evidence for ice transport is prevalent throughout the Late Pliocene. Knies, Mattingsdal, et al. (2014) show an increase in IRD at 3.3 Ma (coincident with MIS M2) at ODP Hole 911A with the weight percent of the 0.1 to 1 mm fraction reaching 14% (Figure 5). During the mPWP, there are small quantities of IRD (< 2% wt) at this site (Knies, Mattingsdal, et al., 2014). In ODP Hole 907A a maximum in IRD is seen at 3.3 Ma reaching 1,746 grains per gram sediment (>125 μm; Fronval & Jansen, 1996), while during the mPWP the number of IRD grains per gram varies from close to 0 to 939 with higher quantities occurring during the colder phases of the mPWP (e.g., KM2; Figure 5; Fronval & Jansen, 1996). In ODP Hole 642B, IRD is present throughout the Late Pliocene; however, there is no distinct increase at MIS M2 (maximum 5 grains per gram) and IRD quantities are generally lower than throughout the mPWP (mPWP quantities reach up to 60 grains per gram at KM4 (>150 μm; Bachem et al., 2016)).

The new occurrence data presented in this study support the presence of IRD throughout the Late Pliocene interval. In addition, the occurrence data documents a consistent presence of IRD at ODP Hole 909C (Figure 5).

## 4 Discussion

The results of this paper have combined modeled iceberg trajectories and the compilation of IRD data to provide further detail of circum‐Nordic Seas ice masses during the mPWP and the MIS M2.

Modeled iceberg trajectories reaching Hole 911A during the M2 scenario are shown to have come from NSVAL. Modeled arctic icebergs (ELLES, ENOVA, and ESEV) also come very close and so are plausible, if less likely, sources. Modeled icebergs from the southwest of Svalbard (SVAL) do not appear to cross Hole 911A during the M2 scenario which are moved westward in the stronger cyclonic gyre created by the subaerial Barents Sea (Figure 4 and Table 3). It is also possible that Hole 911A is covered with perennial sea ice during the M2 scenario according to the model climatology (Figure 3). An increase in IRD (Knies, Mattingsdal, et al., 2014) is seen at Hole 911A during the MIS M2 (Figure 5), an increase that corresponds to Zr/K ratios (Knies, Mattingsdal, et al., 2014). With the nearest point source for zirconium being northern Svalbard (Ottesen et al., 2010), both iceberg trajectory and proxy data favor the same NSVAL source area for IRD deposited at Hole 911A during M2.

**Table 3:** Summary of Iceberg Trajectories From Seeding Locations Which Reach ODP Sites During the M2, mPWP_STD_, and mPWP_ALT_ Climate Scenarios

ODP site	M2	mPWP_STD_	mPWP_ALT_
911A	North Svalbard	West Svalbard	Bear Island Vøring Plateau West Svalbard
909C	North Svalbard West Svalbard	Bear Island West Svalbard	Bear Island Norwegian Channel Vøring Plateau West Svalbard
907A	North Svalbard Eastern Severnaya Zemlya Ellesmere Island East Novaya Zemlya Wandell Sea Nord Morris Jessup Bear Island Vøring Plateau West Svalbard	Bear Island Norwegian Channel Vøring Plateau	North Svalbard (2) Wandell Sea Nord Morris Jessup East Iceland Bear Island Norwegian Channel Vøring Plateau West Svalbard
642B	Bear Island Vøring Plateau	Norwegian Channel Vøring Plateau	Norwegian Channel Vøring Plateau

*Note*. ODP = Ocean Drilling Program. M2 = Marine Isotope Stage M2; mPWP_STD_ = standard mid‐Piacenzian Warm Period; mPWP_ALT_ = mid‐Piacenzian Warm Period scenario with an altered paleogeography.

During the mPWP_STD_ scenario, modeled icebergs seeded from SVAL reached Hole 911A (Figure 4 and Table 3). During the mPWP_STD_ scenario, colder modeled SSTs existed to the north and west of Svalbard than are found in the rest of the Nordic Seas enabling the persistence of icebergs here (Figure 2). Icebergs modeled from SVAL are picked up by the West Spitsbergen Current and transported north onto the Yermak Plateau (Figure 4). During the mPWP_ALT_ scenario, icebergs seeded from SVAL, VORP, and BEAR reach Hole 911A (Figure 4 and Table 3). During the mPWP_ALT_ scenario, colder modeled air temperatures are found along the western margin of the subaerial Barents Sea. Modeled icebergs from VORP and BEAR are transported north in the Norwegian Current and West Spitsbergen Current before reaching the Yermak Plateau and Hole 911A (Figure 4).

In the mPWP IRD is still present at Hole 911A; however, smaller values are indicated (Figure 5) as expected during a warmer period (Knies, Mattingsdal, et al., 2014). The lower values could also be an artifact of increased deposition of background marine sediment instead of less IRD reaching the site in a warm climate. However, the sedimentation rate in Hole 911A decreased from 8 cm/ka to 6 cm/ka between M2 and mPWP, and the lower weight percent of the 0.1 to 1 mm fraction during mPWP can therefore not be an artifact of increased background deposition. The lower amounts of IRD seen in the data through the mPWP are furthermore consistent with modeled trajectories. The low IRD supply has been used as an argument against presence of glacial ice close to the coastline of Svalbard but corroborates existence of small‐scale mountain glaciers (Knies, Mattingsdal, et al., 2014). The site is seasonally covered by sea ice in the model; however, it would be impossible to tell whether the IRD has been entrained by sea ice or glaciers without further analysis by scanning electron microscope to search for microstructures like striations (Gomez et al., 1988).

During the M2 scenario, modeled icebergs seeded from south‐western Svalbard (SVAL) and northern Svalbard (NSVAL) in the Arctic Ocean reach Hole 909C (Figure 4 and Table 3). Icebergs modeled from the south do not reach this site during the cold MIS M2 due to the increased cyclonic gyre in the Nordic Seas which transport them westward before they reach Hole 909C. During the M2 scenario, icebergs from the Arctic would be impeded by sea ice; however, SVAL icebergs would still reach the site. During the winter of the M2 scenario, the site is covered with sea ice (Figure 3).

During the mPWP_STD_ scenario, modeled iceberg trajectories from SVAL still reach this site, as well as those seeded from Bear Island in the Barents Sea (Figure 4 and Table 3). Although warm Atlantic waters are transported northward along the eastern margin of the Nordic Seas by the Norwegian Current (Figure 2), the fast flow allows rapidly melting icebergs to travel some distance (Figure 4). The survival of modeled icebergs seeded at Bear Island suggests that if ice could survive on the land that has subsequently been eroded to form the Barents Sea, then this could be a potential source for Hole 909C IRD (Figure 4). Sea ice is, again, a factor at this site with winter maximum sea ice in the mPWP_STD_ scenario potentially preventing all icebergs reaching this site (Figure 3). During the mPWP_STD_ scenario summer minimum, sea ice is still present at a lower percentage concentration; however, the iceberg trajectories of seeding locations to the west (SVAL and BEAR) appear to reach this site (Figure 4). The modeled iceberg trajectories of Arctic seeding locations do not reach this site in the mPWP_STD_ scenario. In the mPWP_ALT_ scenario, icebergs are prevented from reaching this site due to the sea ice cover in the winter maximum (Figure 3); however, during the summer minimum, again, modeled trajectories from the west Svalbard and Bear Island (SVAL and BEAR) along with those from the Norwegian coast (NORC and VORP) reach this site (Figure 4 and Table 3).

Hole 909C shows presence of IRD in all samples analyzed (Figure 5). The modeled trajectories indicate a predominant source from Svalbard. We only have presence/absence data from this site and no information about variability in amounts or provenance of the IRD deposited. Therefore, the data give no further constraints on which of the seeding locations are more likely. Again, it is impossible to tell whether the quartz grains were transported by seasonal sea ice indicated by the model to reach the site, or icebergs, without further analysis of individual IRD grains.

In the M2 scenario, modeled icebergs from Norway (VORP), Bear Island (BEAR), Svalbard (SVAL and NSVAL), the Arctic (ELLES, ESEV, and ENOVA) and northeast Greenland (MORRIS, NORD, and WAND) all reach ODP Hole 907A (Figure 4 and Table 3) aided by the stronger cyclonic coastal currents around the Nordic Seas. This is despite the fact that throughout the M2 scenario, the central Nordic Seas is up to 4°C warmer than present. Winds, in conjunction with the ocean currents, aid icebergs reaching this site particularly from the Fram Strait. Sea ice during both the winter maximum and summer minimum could complicate the IRD signal at this site (Figure 3) in the M2 scenario.

In the IRD record from Hole 907A, an increase is clearly seen during the cold MIS M2 (Figure 5). The modeled iceberg trajectories are consistent with the increased IRD at Nordic Seas Hole 907A during the MIS M2. There is an increase in the meltwater fractions reaching Hole 907A during the MIS M2, and there are more potential source regions. Logically, either of these factors increase IRD at Hole 907A. However, the increase could also be a result of a combination of both of these factors and not necessarily one or the other.

In the mPWP_STD_ scenario, only modeled icebergs from Norway (NORC, VORP, and BEAR), where no ice would be expected during the warmest phases of the Pliocene (Panitz et al., 2016), reach Hole 907A. Modeled icebergs transported in the EGC, even in the easternmost flow, fail to pass over the site. However, in the simulation with altered paleogeography, modeled icebergs from Norway, along with those from the seeding locations of Svalbard (NSVAL and SVAL), northern Greenland (WAND, NORD, and MORRIS), and Iceland (EICE) are transported to this site (Figure 4 and Table 3).

The IRD record from Hole 907A shows a reduction in the amount of IRD during the mPWP relative to the MIS M2 (Figure 5). According to the LR04 stack, this spans some of the strongest warming events during this time, KM5 and KM3 (Haywood, Hill, et al., 2013; Raymo et al., 2011) when least IRD is deposited at Hole 907A (Fronval & Jansen, 1996) (Figure 5). The significant decrease in deposited IRD during the mPWP warm phases may indicate the loss of marine‐terminating glaciers due to the warming extremes from the MIS M1 through to KM3, although other potential long term consequences of warming, for example, ocean circulation changes, cannot be ruled out. During the mPWP, the amount of IRD increases somewhat between 3.10 and 3.15 Ma (Fronval & Jansen, 1996), which is consistent with a cold phase identified as KM2 (Lisiecki & Raymo, 2005).

It is not thought that icebergs carrying sufficient IRD or being large enough to survive crossing the warm (Bachem et al., 2016) Nordic Seas to Hole 907A could be produced by locations such as the Norwegian coast or the western edge of the Barents Sea in this climate and yet IRD is present. New sea ice reconstructions from Site 907 document seasonal sea ice, with occasional sea ice free conditions, throughout the MIS M2 to mPWP (Clotten et al., 2018). This seasonal sea ice is argued either to be produced at site or transported to the site via the EGC. No provenance data exist for the time interval investigated here; however, for the Holocene, the most likely provenance of IRD deposited at Hole 907A is argued to be the east coast of Greenland, north of Scoresby Sund, based on Pb isotopes (White et al., 2016). Warm Norwegian Sea SSTs, an equal to or stronger zonal temperature gradient in the Nordic Seas than during the present interglacial (Panitz et al., 2017), seasonal sea ice in the Iceland Sea, and a Greenland source area for IRD reaching Hole 907A during the Holocene support the altered paleogeography scenario of Hill (2015) where IRD sources from Northeast Greenland (WAND, NORD, and MORRIS), Iceland (EICE), and Svalbard (SVAL and NSVAL2) reach Hole 907A.

During the M2, three seeding locations from Norway (NORC, VORP, and BEAR) are the only ones modeled which produce icebergs reaching Hole 642B (Figure 4 and Table 3). During the M2 scenario, the modeled SST anomaly along the Norwegian coast are up to 9°C cooler than the central Nordic Seas. The coastal waters of Norway are up to approximately 5°C cooler than present. Any iceberg entrained into these waters would potentially persist and move north and westward in the Norwegian Current. In the model, the inner Vøring Plateau is subaerial and so any icebergs seeded from these locations are likely to move overhead of Hole 642B. Stronger winds from the Norwegian coast effectively combine forces with the North Atlantic Current moving icebergs north over the site before heading westward. Both the Norwegian coast seeding locations and Hole 642B are sea ice free during summer and winter months (Figure 3 and Table 3).

Modeled iceberg trajectories reaching this site during the mPWP_STD_ scenario come from Norway (NORC and VORP; Figure 4 and Table 3). The SSTs near both of these seeding locations is 3°C to 4°C higher than present. The currents near these locations are strong, but the warm waters would quickly melt any icebergs caught up in the Norwegian Current. However, as winds move westward off the Norwegian coast and play a significant secondary role in determining the direction icebergs move (Matsumoto, 1997), it is possible that icebergs could have been “pushed” out to the edge of the Vøring Plateau from these two seeding locations. During both summer and winter, these two locations are unimpeded by sea ice (Figure 3). The modeled mPWP_ALT_ scenario shows NORC and VORP, again, to be the only two seeding locations with iceberg trajectories reaching Hole 642B.

The IRD records for Hole 642B show variability in the amount of icebergs reaching this site. Contrasting the observations from the other sites, the MIS M2 is not characterized by a larger IRD peak for Hole 642B (Figure 5). A minor hiatus over the most extreme part of MIS M2 has been suggested, or MIS M2 was less extreme here than other places (Risebrobakken et al., 2016). In the occurrence data, only one sample showed an absence of IRD (Figure 5), while in the >150 μm fraction record, Bachem et al. (2016) found five samples with no IRD (3.269 Ma, 3.260 Ma, 3.226 Ma, 3.223 Ma, and 3.218 Ma) consistent with the warmer phases of the mPWP (MIS M1 through to KM5). Slightly more IRD is found in the colder phases of the mPWP than in the warm phases (Figure 5).

The lack of any modeled meltwater fraction at Hole 642B from Arctic, Greenlandic or Icelandic source regions is also in line with the lower count values of IRD found in the IRD data records.

The modeled iceberg trajectory results show Norway as a potential source, which is not easily reconcilable with evidence from paleoenvironmental reconstruction.

Pollen records from Hole 642B suggest cool temperate forest existed during the mPWP on the coast of Norway (Panitz et al., 2016). This is evidence for a climate too warm for marine‐terminating glaciers to exist here. This prompts the questions as to how IRD is found during this time period at Hole 642B. The IRD could not have come from sea ice deposition as modeled sea ice during the mPWP does not reach the Norwegian coast and both modeled (Figure 2) and reconstructed (Bachem et al., 2016) SSTs are too warm. For sea ice to form, colder temperatures are required than temperatures needed for sustaining a marine‐terminating glacier (Jansen & Sjøholm, 1991). The absolute number of IRD grains deposited at Hole 642B is very small, even for maximum peaks, and are of the order of magnitude seen at the Vøring Plateau during the last interglacial (Figure 5), a time when Norwegian glaciers did not reach the coast (Mangerud, 2004). Bachem et al. (2016) argued for a Greenland source for the IRD, while Jansen and Sjøholm (1991) suggested that the IRD data from Hole 642B reflect a “less extensive” glaciation in Scandinavia in the form of mountain, valley or fjord glaciers, or from glaciers from Greenland and Svalbard. Unfortunately, no provenance information exists from any of the Hole 642B records. Furthermore, there are challenges related to separating the Norwegian and Greenland sources of IRD by provenance. Greenland and Norway were of the same continental crust up until Tertiary time when continental rifting opened the northern North Atlantic (Mosar et al., 2002). Due to the similar geological history, there is quite some overlap between potential source regions in the Pb‐Pb space (Bailey et al., 2012).

At face value, our modeling results agree that iceberg trajectories from Greenland or Svalbard do not cross Hole 642B. While our model results agree with the Jansen and Sjøholm (1991) assessment, the very low abundance of IRD in the records presented, combined with the new terrestrial environmental record of Panitz et al. (2016) lead us to conclude that the possibility of marine‐terminating glaciers on Scandinavia at this time is low.

This research has highlighted the need for further study of these sites as many questions remain outstanding. How does IRD reach Hole 642B on the Vøring Plateau during a warmer climate than present, when at present there are no icebergs? Where does the IRD come from that reaches Hole 907A? Is the IRD at Sites 909C and 911A deposited by icebergs or sea ice? The research done here relies on the assumptions that the ocean circulation in the model framework is correct and that IRD is transported to a site by icebergs and sea ice only. It also assumes that SSTs in the Nordic Seas are warmer than present (Bachem et al., 2016). There are several points to consider here. First, Sites 907A, 909C, and 911A are seasonally affected by sea ice (Figure 3), and so to establish the presence or absence of icebergs, quartz grains need to be tested for microstructures which give a better picture of the mode of transport (Gomez et al., 1988; Menzies et al., 2013, and references therein; St. John et al., 2015). Secondly, proxy evidence from the region varies in confidence. Evidence from terrestrial sources (e.g., Bennike et al., 2002; Panitz et al., 2016; Salzmann et al., 2009, 2011) give an indication that temperatures are higher than present in the region during the mPWP. Proxy evidence of SSTs from marine sources exist all show warmer than present temperatures (Bachem et al., 2016; Clotten et al., 2018; Knies, Cabedo‐Sanz, et al., 2014; Robinson, 2009; Schreck et al., 2013), with a few exceptions from Site 907, where summer SSTs slightly colder than today are seen during MIS M2, KM5b, MK2, K1 and G20 (Clotten et al., 2018). There is however, a large range between the reconstructed warming in the Nordic Seas region. Not only does the evidence from these proxy data suggest that land temperatures are too warm to sustain a calving front on Scandinavia, Barents Sea, and Iceland, but also that if it was possible, SSTs would be sufficiently warm to melt icebergs in a very short period of time using the assumption that icebergs would be small. The proxy data suggest that seeding locations such the Norwegian coast (NORC and VORP), Bear Island and Iceland would not produce icebergs. Therefore, IRD should not be found at ODP Sites 642B or 907A according to the ocean circulation in the standard PlioMIP mPWP model, although we accept that this may be a model‐dependent result. Even if icebergs were produced from these sites, the question arises: would they be sufficiently large enough to cross the Nordic Seas to Hole 907A using the standard PlioMIP paleogeography and climate conditions? Using the altered paleogeography of Hill (2015), icebergs sourced from north Svalbard and northeast Greenland provide more plausible source locations for IRD to reach Hole 907A.

## 5 Conclusion

Iceberg modeling and IRD data have, for the first time, been combined to provide clues to the extent of the ice margins surrounding the Nordic Seas. This novel combination shows the potential source locations from which icebergs reach ODP sites around the Nordic Seas. ODP sites around Svalbard receive icebergs throughout the mPWP. These IRD records are likely to be primarily sourced from Svalbard itself, although other Arctic icebergs may contribute.

In the M2 and mPWP_ALT_ climate scenarios, Hole 907A receives icebergs that are entrained in the eastern margins of the EGC. However, in the mPWP_STD_ scenario the current does not deliver modeled icebergs to Hole 907A. This means that only seeding locations from Norway reach this site which seems unlikely to be ice covered during the warmest parts of the Pliocene. This suggests that the changes in circulation associated with simulations incorporating altered paleogeography are important for the delivery of icebergs to 907A. The greater number of seeding locations with trajectories modeled to reach Hole 907A supports the evidence of higher amounts of IRD at this hole in contrast to that of Hole 642B. Under the mPWP_ALT_ scenario, icebergs from Svalbard, northern Greenland, and the east coast of Iceland are plausible IRD sources. Of these sources only northern Greenland is maintained in the M2 scenario. Abundance of IRD at Hole 642B is lower prior to the MIS M2 glacial period and at the MIS M1 warm peak. Combined with the Svalbard records, this suggests a consistent Svalbard source with northern Greenland icebergs swamping these from the MIS M2, apart from the very warmest period. Hole 642B receives icebergs only from the coast of Norway in the M2, mPWP_STD_, and mPWP_ALT_ scenarios. No other seeding locations have modeled icebergs which reach this site during the warmer‐than‐modern mPWP, meaning the enigma of the persistent IRD off the coast of Norway remains, since a Norwegian source is inconsistent with present understanding of the Late Pliocene Norwegian climate.

Overall, combining IRD records and iceberg modeling suggests that ice persists on Svalbard throughout the climate changes of the mPWP. It also suggests that ice is present on the northeast Greenland coast for much of the mPWP but that these locations do not produce icebergs or, at least, not sufficiently large enough icebergs, to reach the ODP Sites before melting during the warmest periods. There are several locations from which icebergs are seeded which reach each location in different climate scenarios. The altered paleogeography of Hill (2015) is important as this modeled scenario provides iceberg sources from different and more plausible locations. This illustrates the necessity of further studies of IRD from these sites to analyze the extent of ice surrounding the Nordic Seas.
